# Use of Telemedicine for Buprenorphine Inductions in Patients With Commercial Insurance or Medicare Advantage

**DOI:** 10.1001/jamanetworkopen.2021.42531

**Published:** 2022-01-06

**Authors:** Benjamin A. Barsky, Alisa B. Busch, Sadiq Y. Patel, Ateev Mehrotra, Haiden A. Huskamp

**Affiliations:** 1 Interfaculty Initiative in Health Policy, Harvard University, Cambridge, Massachusetts; 2 Department of Health Care Policy, Harvard Medical School, Boston, Massachusetts; 3 Hospital Administration, McLean Hospital, Belmont, Massachusetts; 4 Division of General Medicine, Beth Israel Deaconess Medical Center, Boston, Massachusetts; 5 OptumLabs, Eden Prairie, Minnesota

## Abstract

This cross-sectional study examines the use of telemedicine for buprenorphine inductions among individuals with commercial insurance or Medicare Advantage during the temporary repeal of the Ryan Haight Act requirement of in-person evaluation before prescribing buprenorphine during to the COVID-19 pandemic.

## Introduction

Because it constrains telemedicine use for opioid use disorder (OUD), many have advocated for the repeal of the Ryan Haight Act requirement that clinicians conduct an in-person evaluation before prescribing buprenorphine.^1,2^ The SUPPORT Act of 2018 requires the Drug Enforcement Administration (DEA) to create a regulatory pathway for buprenorphine prescribing via telemedicine, but the DEA has yet to do so. Concerns remain at the DEA about a possibly greater diversion risk when clinicians prescribe via telemedicine.^3^

Early in the COVID-19 pandemic, regulators waived the restriction of the Ryan Haight Act to expand access to OUD treatment.^4^ This temporary waiver allowed us to study telemedicine use for buprenorphine inductions in a commercially insured population.

## Methods

This cross-sectional study used deidentified commercial and Medicare Advantage claims data from the OptumLabs Data Warehouse and identified buprenorphine inductions from January 1, 2020, to April 30, 2021 (eAppendix in the Supplement). Harvard Medical School’s institutional review board exempted this study and waived the requirement for informed consent under 45 CFR 46.104(d)(4)(2). We performed statistical analyses in SAS, version 9.4 (SAS Institute Inc), and followed the Strengthening the Reporting of Observational Studies in Epidemiology (STROBE) reporting guideline for cross-sectional studies.

During the pandemic period of April 1, 2020, to April 30, 2021, we described the monthly rate of buprenorphine inductions via telemedicine and, using multivariable logistic regression, compared patient characteristics of those receiving telemedicine vs in-person inductions. We focused on clinical severity and/or complexity markers (eg, severe mental illness diagnosis, OUD-related emergency department visit, recent benzodiazepine fill), and socioeconomic characteristics potentially associated with difficulty accessing or using technology (eg, older age, rural residence, residing in a lower-income county; variables are defined in the eAppendix in the Supplement). Statistical significance was defined as a 95% CI excluding 0.

## Results

Among the 2703 patients who received a buprenorphine induction between April 1, 2020, and April 30, 2021, inductions for 377 patients (13.9%) were via telemedicine (mean [SD] age, 46.2 [14.8] years; 161 [42.7%] were women and 216 [57.3%] were men). The other 2326 patients (86.1%) received the inductions in person (mean [SD] age, 49.7 [15.9] years; 1019 [43.8%] were women and 1307 [56.2%] were men). Telemedicine inductions increased from no inductions in January 2020 (prepandemic) to 42 in April 2020 (constituting 21.2% of monthly inductions), then decreased to 14 inductions by April 2021 (constituting 7.9% of monthly inductions) (Figure).

**Figure.**
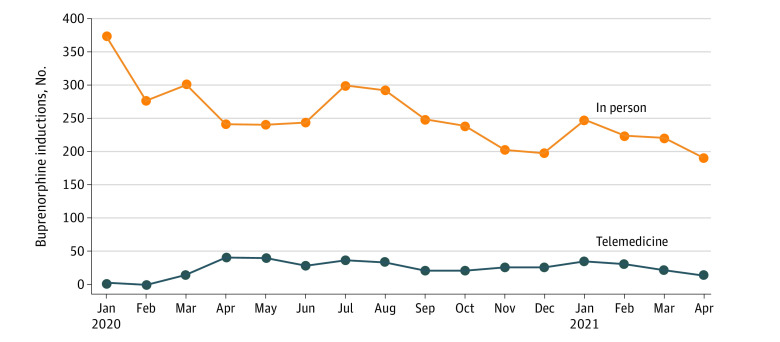
Absolute Number of Buprenorphine Inductions Conducted via Telemedicine Among Commercial and Medicare Advantage Enrollees, January 1, 2020, Through April 30, 2021 We identified 3638 inductions during this 16-month study period, 391 (10.8%) of which occurred via telemedicine.

We found no statistically significant differences between patients receiving telemedicine inductions vs in-person inductions in clinical severity and/or complexity markers, nor in whether they had a visit with the induction provider before the induction (Table). Patients 70 years or older were less likely to have a telemedicine induction compared with those aged 30 to 49 years (average marginal effect difference, −7.8%; 95% CI, −12.5% to −3.2%). Patients living in a county in a higher quartile of median household income were more likely to have a telemedicine induction vs those in the lowest quartile (eg, average marginal effect difference for first vs fourth quartile, 6.5%; 95% CI, 2.4%-10.7%).

**Table.** Characteristics of Patients Undergoing Telemedicine vs In-Person Inductions Among Commercial and Medicare Advantage Enrollees, April 1, 2020, through April 30, 2021

Characteristic	Inductions, No. (%)		Average marginal effect on having a telehealth induction, % point change (95% CI)^a^	
	Telemedicine (n = 377)	In-person (n = 2326)	Unadjusted	Adjusted
Age group, y				
30-49	158 (41.9)	824 (35.4)	1 [Reference]	1 [Reference]
15-29	61 (16.2)	293 (12.6)	1.1 (−3.4 to 5.7)	−1.2 (−5.4 to 3.0)
50-69	140 (37.1)	972 (41.8)	−3.5 (−6.5 to −0.5)	−2.2 (−5.8 to 1.3)
≥70	18 (4.8)	237 (10.2)	–9 (−12.9 to −5.1)	−7.8 (−12.5 to −3.2)
Documented sex				
Female	161 (42.7)	1019 (43.8)	1 [Reference]	1 [Reference]
Male	216 (57.3)	1307 (56.2)	0.5 (−2.1 to 3.2)	−0.8 (−3.5 to 1.9)
Urban/rural residence^b^				
Nonrural	307 (81.4)	1766 (75.9)	1 [Reference]	1 [Reference]
Rural	70 (18.6)	560 (24.1)	−3.7 (−6.6 to −0.8)	0.1 (−3.5 to 3.7)
Insurance type				
Commercial	235 (62.3)	1190 (51.2)	1 [Reference]	1 [Reference]
Medicare Advantage	142 (37.7)	1136 (48.8)	−5.4 (−8.0 to −2.8)	−0.8 (−4.6 to 2.9)
Census region				
South	120 (31.8)	1177 (50.6)	1 [Reference]	1 [Reference]
Midwest	84 (22.3)	465 (20)	6.0 (2.6 to 9.4)	4.5 (0.9 to 8.2)
Northeast	68 (18)	288 (12.4)	9.8 (5.5 to 14.2)	6.8 (2.3 to 11.3)
West	105 (27.9)	396 (17)	11.7 (7.8 to 15.6)	9.2 (5.2 to 13.3)
Clinical risk factors^c^				
Severe mental illness diagnosis	34 (9)	193 (8.3)	1.1 (−3.7 to 6.0)	0.0 (−4.8 to 4.7)
ED visits with OUD as primary diagnosis	12 (3.2)	44 (1.9)	7.6 (−3.2 to 18.5)	3.3 (−6.0 to 12.7)
Prior encounter with induction provider	109 (28.9)	704 (30.3)	−0.8 (−3.6 to 2.0)	−1.4 (−4.3 to 1.5)
Non-OUD substance use disorder diagnosis	79 (21)	415 (17.8)	2.5 (−1.0 to 6.0)	−1.1 (−6.8 to 4.6)
Prior benzodiazepine prescription fill	45 (11.9)	214 (9.2)	3.8 (−1.0 to 8.6)	1.0 (−3.5 to 5.5)
Moderate or severe OUD diagnosis	60 (15.9)	291 (12.5)	3.6 (−0.6 to 7.8)	2.7 (−4.7 to 10.2)
Socioeconomic risk factors^d^				
County proportion of White individuals, quartile (%)				
Low (<42.3)	35 (9.3)	235 (10.1)	1 [Reference]	1 [Reference]
2 (42.3-69.7)	119 (31.6)	667 (28.7)	2.2 (−2.5 to 6.9)	2.5 (−2.3 to 7.3)
3 (69.8-85.8)	94 (24.9)	639 (27.5)	−0.1 (−4.8 to 4.5)	−1.2 (−5.9 to 3.5)
High (>85.8)	129 (34.2)	785 (33.7)	1.2 (−3.4 to 5.7)	1.1 (−3.8 to 6.0)
County median household income, quartile ($)				
1 (<41 042)	76 (20.2)	823 (35.4)	1 [Reference]	1 [Reference]
2 (<41 042-52 197)	95 (25.2)	563 (24.2)	6.0 (2.7 to 9.2)	5.0 (1.4 to 8.5)
3 (<52 198-69 240)	103 (27.3)	480 (20.6)	9.2 (5.6 to 12.8)	5.8 (1.9 to 9.6)
4 (>69 240)	103 (27.3)	460 (19.8)	9.8 (6.2 to 13.5)	6.5 (2.4 to 10.7)

Abbreviations: ED, emergency department; OUD, opioid use disorder.

^a^ The average marginal effects under our unadjusted and adjusted models capture the mean change in the outcome probability when a risk factor variable changes from 0 (ie, the reference category) to 1 (ie, a nonreference category). As such, under our adjusted model, we find, for example, that the probability of receiving a telehealth induction decreases by 7.8% when moving from a patient aged 30 to 49 years vs a patient aged 70 years or older.

^b^ Rural-urban communing area under the 4-category US Census designation.

^c^ Clinical risk factor variables were identified using claims for the period between 8 and 180 days before the index buprenorphine treatment.

^d^ Enrollee county-level indicators for race and income from the 2020 US Census. We divided county-level measures into quartiles for ease of interpretation.

## Discussion

The findings of this cross-sectional study suggest that, after relaxation of the Ryan Haight Act’s restriction, 13.9% of buprenorphine inductions were via telemedicine. Contrary to expectations,^5^ we saw no difference in telemedicine use among patients with markers of greater clinical severity and/or complexity. Instead, we observed fewer telemedicine inductions among older adults and individuals in lower-income counties. These demographic groups may have lacked the necessary digital literacy or technology to use telemedicine.

Limitations of this study are that these analyses include only individuals from 1 large insurer, limiting their generalizability to other insured (eg, Medicaid) and uninsured populations, and that the results were subject to ecologic bias given the inclusion of county-level demographic variables.

Our results may allay concerns stated by the DEA that relaxing the in-person evaluation requirement of the Ryan Haight Act may increase buprenorphine diversion. Even at its peak during the pandemic, the proportion of inductions via telemedicine (21.2%) was much lower than rates of telemedicine use for other behavioral health conditions (eg, 56% of behavioral health visits in December 2020).^6^ The relatively lower use of telemedicine means that opportunities for diversion are limited. Of note, we must acknowledge that no clear evidence shows that telemedicine increases diversion risk. The availability of telemedicine may in fact expand access to treatment, which is crucial if we want to maximize pathways of care for patients with OUD, particularly given the ongoing opioid crisis.

## Notes

Supplement. **eAppendix.** Identifying Study Cohort, Buprenorphine Inductions, and Markers of Clinical Severity
