# Supplementary material for: Use of Telemedicine for Buprenorphine Inductions in Patients With Commercial Insurance or Medicare Advantage
Source: JAMA Netw Open. 2022 Jan 6;5(1):e2142531. doi: 10.1001/jamanetworkopen.2021.42531 (PMC8739765; doi:10.1001/jamanetworkopen.2021.42531)
Supplement: Supplement. — eAppendix. Identifying Study Cohort, Buprenorphine Inductions, and Markers of Clinical Severity [file jamanetwopen-e2142531-s001.pdf]

Supplemental Online Content

Barsky BA, Busch AB, Patel SY, Mehrotra A, Huskamp HA. Use of telemedicine for buprenorphine inductions in patients with commercial insurance or Medicare Advantage. *JAMA Netw Open.* 2022;5(1):e2142531. doi:10.1001/jamanetworkopen.2021.42531

eAppendix. Identifying Study Cohort, Buprenorphine Inductions, and Markers of Clinical Severity

This supplemental material has been provided by the authors to give readers additional information about their work.

© 2022 Barsky BA et al. *JAMA Network Open.*

eAppendix. Identifying Study Cohort, Buprenorphine Inductions, and Markers of Clinical Severity

Identification of study cohort and buprenorphine inductions:

Study cohort: We required continuous enrollment in medical, behavioral health, and pharmacy benefits 180 days before the index buprenorphine treatment (see definition below).

Buprenorphine inductions: We defined telemedicine and in-person buprenorphine inductions using the following three-pronged process.

1. We identified the first buprenorphine fill or office administrations (i.e., index buprenorphine treatment) during the period January 2020 through April 2021.

   a. Buprenorphine fills were defined as pharmacy claims for generic and brand versions of buprenorphine or, buprenorphine/naloxone, whether oral, intramuscular or implant versions. Pharmacy claims for buprenorphine without naloxone required an OUD diagnosis code (ICD-10 code F11.xxx) in the primary or secondary field on associated face-to-face outpatient claims.

   b. Buprenorphine administrations were defined by claims for Healthcare Common Procedure Coding System (HCPCS) and Current Procedural Terminology (CPT) codes for injections, implants or facility-administered medications: G0516, G0518, H0033, J0571-J0575, J0592, Q9991, Q9992, J0570-J0575, 11981, 11983, and 96372. We required an OUD diagnosis code in the primary or secondary field for a subset of these codes because they are not specific to buprenorphine administration: G0516, G0518, H0033, J0571, J0592, 11981, and 11983.

2. We required a 90-day clean period—that is, beneficiaries could not have received buprenorphine prescriptions or office-based administrations in the 90 days prior to the index buprenorphine treatment.

3. To determine whether the induction was conducted via telemedicine vs. in-person, we first linked index buprenorphine treatments with outpatient visits occurring 7 days prior to or up to 3 days after the index buprenorphine treatment.

We defined an outpatient visit as claims with a visit for any one of the following outpatient CPT and HCPCS codes: 00104, 0901, 0911, 9424, 9426, 9427, 90791, 90792, 90801, 90802, 90804, 90805, 90806, 90807, 90808, 90809, 90810, 90811, 90812, 90813, 90814, 90815, 90820, 90832, 90833, 90834, 90835, 90836, 90837, 90838, 90839, 90840, 90842, 90843, 90844, 90845, 90846, 90847, 90848, 90849, 90853, 90855, 90857, 90862, 90865, 90867, 90868, 90869, 90870, 90871, 90875, 90876, 90880, 90900, 90901, 90902, 90904, 90906, 90908, 90910, 96372, 97003, 97004, 98960, 98961, 98962, 99058, 99078, 99201, 99202, 99203, 99204, 99205, 99211, 99212, 99213, 99214, 99215, 99241, 99242, 99243, 99244, 99245, 99341, 99342, 99343, 99344, 99345, 99347, 99348, 99349, 99350, 99382, 99383, 99384, 99385, 99386, 99387, 99392, 99393, 99394, 99395, 99396, 99397, 99401, 99402, 99403, 99404, 99404, 99408, 99409, 99411, 99412, 99420, 99490, 99495, 99496, 99510, 0359T, 0360T, 0361T, 0362T, 0363T, 0364T, 0365T, 0366T, 0367T, 0368T, 0369T, 0370T, 0371T, 0372T, 0373T, 0374T, G0071, G0072, G0073, G0074, G0075, G0076, G0077, G0078, G0079, G0080, G0081, G0082, G0155, G0175, G0351, G0396, G0397, G0442, G0443, G0463, G0466, G0467, G0469, G0470, G0505, G0507, G0513, G0514, G0515, G2069, G2074, H0001, H0002, H0004, H0005, H0006, H0007, H0014, H0016, H0020, H0022, H0023, H0028, H0029, H0031, H0033, H0034, H0036, H0037, H0038, H0039, H0040, H0046, H0049, H0050, H1011, H2000, H2001, H2010, H2011, H2013, H2014, H2015, H2016, H2017, H2018, H2019, H2020, H2021, H2022, H2023, H2024, H2025, H2026, H2027, H2028, H2029, H2030, H2031, H2032, H2033, H2037, H5010, H5020, H5025, H5030, H5220, H5230, H5240, H5299, M0064, Q3014, S3005, S9110, S9127, S9454, S9482, S9484, S9485, T1006, T1006, T1007, T1011, T1012, T1015, T1016, T1017, T1018, T1023, T1024, T1025, T1026, T1027, T1040, T1041, T2010, T2011, T2012, T2013, T2014, T2015, T2018, T2019, T2020, T2021, T2022, T2023, T2034, T2036, T2037, Z0001, Z0002, Z0002.

© 2022 Barsky BA et al. *JAMA Network Open.*

Claims for visits delivered via telemedicine had modifier codes (GT, GQ, 95) or were claims for visits with HCPCS codes specific to telemedicine and potentially applicable to OUD treatment (99441–99443, 98966–98968, G2025).

If the index treatment was an office-based administration, we considered the claim for that administration to be the outpatient visit associated with the induction, and this induction was considered to be an in-person induction. To identify the outpatient visit associated with index treatments that were buprenorphine prescription fills, we first looked for outpatient visits with the same provider key on the outpatient visit claim as on the index pharmacy claim. If the enrollee had a single outpatient visit with the same provider key during that -7/+3 day window, that visit was considered the induction visit.

If the enrollee had more than one outpatient visit with the same provider key during that window, the induction was considered to be an in-person induction if any of those visits were conducted in-person; if all of those visits were via telemedicine, it was considered to be a telemedicine induction.

For index treatments with no outpatient visits during the -7/+3-day window with the same provider key as the pharmacy claim, we looked for visits in that window with an OUD diagnosis code in the primary or secondary diagnosis field. If the enrollee had a single visit during this window that met this criterion, that visit was considered the induction visit. If the enrollee had more than one outpatient visit during that window that met this criterion, the induction was considered to be an in-person induction if any of those visits were conducted in-person; if all of those visits were via telemedicine, it was considered to be a telemedicine induction.

Defining markers of clinical severity or complexity:

Serious mental illness (SMI) diagnosis: Whether patients had any inpatient or outpatient claim with a diagnosis of schizophrenia/related psychotic disorder or bipolar disorder diagnosis (ICD-10 code F20.xxx-F31.xxx, F34.0) in any diagnosis field 180 to 8 days before their index buprenorphine treatment.

Emergency department (ED) visits with OUD as primary diagnosis: Whether patients had any emergency department claim (CPT: 90500, 90505, 90510, 90515, 90517, 90520, 90530, 90540, 90550, 90560, 90570, 90580, 99281-99281) with OUD in the primary diagnosis field 180 to 8 days before their index buprenorphine treatment.

Prior encounter with induction provider: Whether patients had any inpatient or outpatient visit with the provider responsible for their buprenorphine induction 180 to 8 days before their index buprenorphine treatment.

Non-OUD substance use disorder diagnosis: Whether patients had any inpatient or outpatient claim with a non-OUD substance use disorder diagnosis (ICD-10 code F10.xxx, F12.xxx-F19.xxx excluding F17.xxx) in any field 180 to 8 days before their index buprenorphine treatment.

Prior benzodiazepine fill: Whether patients filled any prescriptions for benzodiazepine 180 to 8 days before their index buprenorphine treatment. We defined benzodiazepine fills as pharmacy claims for the generic or brand version of alprazolam, chlordiazepoxide/clidinium, clipoxide, chlordiazepoxide, clonazepam, clorazepate, diazepam, estazolam, flurazepam, halazepam, paxipam, lorazepam, oxazepam, prazepam, quazepam, temazepam, triazolam.

Moderate or severe OUD diagnosis: Whether patients had any inpatient or outpatient claim for moderate (ICD-10 code F11.2xx) or severe (ICD-10 code F11.1xx) OUD 180 to 8 days before their index buprenorphine treatment.

© 2022 Barsky BA et al. *JAMA Network Open.*
